# Pilot Investigation of SARS-CoV-2 Variants in the Island of Sicily Prior to and in the Second Wave of the COVID-19 Pandemic

**DOI:** 10.3389/fmicb.2022.869559

**Published:** 2022-04-26

**Authors:** Miguel Padilla-Blanco, Francesca Gucciardi, Annalisa Guercio, Vicente Rubio, Antonina Princiotta, Veronica Veses, Mariangela Terrana, Chirag C. Sheth, Marina Pascual-Ortiz, Elisa Maiques, Giuseppa Purpari, Consuelo Rubio-Guerri

**Affiliations:** ^1^Department of Pharmacy, Faculty of Health Sciences, Universidad CEU Cardenal Herrera, CEU Universities, Valencia, Spain; ^2^Istituto Zooprofilattico Sperimentale della Sicilia “A. Mirri”, Palermo, Italy; ^3^Instituto de Biomedicina de Valencia (IBV-CSIC), CIBER of Rare Diseases (CIBERER-ISCIII), Valencia, Spain; ^4^Department of Biomedical Sciences, Faculty of Health Sciences, Universidad CEU Cardenal Herrera, CEU Universities, Valencia, Spain; ^5^Department of Medicine, Faculty of Health Sciences, Universidad CEU Cardenal Herrera, CEU Universities, Valencia, Spain

**Keywords:** SARS-CoV-2, Sicily (Italy), B.1.160 variant, B.1.177 variant, COVID-19 in autumn 2020

## Abstract

After 2 years of the COVID-19 pandemic, we continue to face vital challenges stemming from SARS-CoV-2 variation, causing changes in disease transmission and severity, viral adaptation to animal hosts, and antibody/vaccine evasion. Since the monitoring, characterization, and cataloging of viral variants are important and the existing information on this was scant for Sicily, this pilot study explored viral variants circulation on this island before and in the growth phase of the second wave of COVID-19 (September and October 2020), and in the downslope of that wave (early December 2020) through sequence analysis of 54 SARS-CoV-2-positive samples. The samples were nasopharyngeal swabs collected from Sicilian residents by a state-run one-health surveillance laboratory in Palermo. Variant characterization was based on RT-PCR amplification and sequencing of four regions of the viral genome. The B.1.177 variant was the most prevalent one, strongly predominating before the second wave and also as the wave downsized, although its relative prevalence decreased as other viral variants, particularly B.1.160, contributed to virus circulation. The occurrence of the B.1.160 variant may have been driven by the spread of that variant in continental Europe and by the relaxation of travel restrictions in the summer of 2020. No novel variants were identified. As sequencing of the entire viral genome in Sicily for the period covered here was restricted to seven deposited viral genome sequences, our results shed some light on SARS-CoV-2 variant circulation during that wave in this insular region of Italy which combines its partial insular isolation with being a major entry point for the African immigration.

## Introduction

In the late December 2019, the novel human-infecting severe acute respiratory syndrome coronavirus 2 (SARS-CoV-2) was reported in Wuhan, Hubei Province, China (ProMED-mail, 2020; Zhu et al., 2020). Since then, this virus spread around the globe, causing a global pandemic, known as coronavirus disease 2019 (COVID-19). Up to 3 February 2022, the WHO (Coronavirus Resource Center, 2021; World Health Organization [WHO], 2022a) recorded > 380 million infections worldwide and almost 5.7 million COVID-19 deaths. The history of this pandemic is punctuated by the emergence of novel viral variants differing from the one described originally, some of which are variants of concern due to their increased infectivity and potential escape from immunity derived from prior infection with earlier variants, or, since January 2021, from vaccination (Worobey et al., 2020; Funk et al., 2021). Variants result from spontaneous mutations in the viral genome that introduce changes in the encoded proteins, particularly on the *spike* (*S*) gene which encodes the spike glycoprotein (S protein), the key mediator of the interaction of the virus with the host cells, leading to infection (Chakraborty et al., 2022). Thus, the monitoring of the emergence of novel viral variants is crucial, as variants can result in changes in viral transmissibility, virulence, antigenicity, and recognition by the adaptive immune system triggered by prior infection or vaccination (Funk et al., 2021). For example, in early 2020, SARS-CoV-2 sequences which included the D614G mutation in the S protein exhibited higher human transmissibility than the original variant of the Wuhan outbreak, resulting in the occurrence of this mutation in all the variants that have circulated afterward (Korber et al., 2020; Volz et al., 2021).

Because of the strict control measures on mobility and social distancing imposed in Europe during the spring of 2020, COVID-19 cases dwindled, and the appearance of new variants was minimized (Hodcroft et al., 2021). Subsequent relaxation of restrictions, including the resumption of travel in the summer of 2020, led to the re-emergence of the disease throughout Europe, with the spread of new variants (Hodcroft et al., 2021). In June 2020, the B.1.177 variant emerged in Spain and spread throughout Europe, becoming in some countries (such as Iceland, Ireland, and Spain), the major circulating variant (Hodcroft et al., 2021). Relative to the early Wuhan isolates, the B.1.177 variant presented variant-defining mutations in the *S*, *nucleocapsid* (*N*), and *ORF10* genes, causing the amino acid changes S:A222V (S protein), N:A220V (nucleocapsid phosphoprotein, abbreviated N protein) and ORF10:V30L (ORF10 protein). The corresponding nucleotide changes were C22227T, C28932T, and G29645T (numbered according to the SARS-CoV-2 reference genome, GenBank Accession Number: NC_045512.2) (Hodcroft et al., 2021). Later variants appearing in Europe during 2020 exhibited other mutations in the S protein, such as the S:S477N mutation of the B.1.160 variant, or, in separate clusters, of other S protein mutations, such as D80Y, S98F, and N439K (Hodcroft et al., 2021). None of these mutations seemed to importantly increase viral transmission or virulence relative to the B.1.177 variant, or to cause evasion from antibodies (or later on from vaccines) (Hodcroft et al., 2021; Xie et al., 2021). Therefore, the incorporation of these changes and the temporal success of some of them appear to be a consequence of genetic bottlenecks created by the low circulation of the SARS-CoV-2 virus after the period of generalized movement restriction throughout Europe (Hodcroft et al., 2021).

In any case, as already indicated, the S protein is of particular interest concerning the incorporation of changes and development of variants, as it is largely responsible for viral attachment to the host cell *via* interaction with the cellular receptor for this virus, the angiotensin-converting enzyme 2 (ACE2) (Jackson et al., 2022). Mutations leading to alterations in the amino acid sequence of the S protein can strongly modify viral fitness (Lan et al., 2020). This is exemplified by the B.1.1.7, B.1.351, and P.1 variants, which presented eight, six, and ten mutations in their S protein, respectively (Gómez et al., 2021), with concomitant 71% increased transmissibility in the case of B.1.1.7 (Bian et al., 2021), while the B.1.351 and P.1 variants decreased the effectivity of therapeutic antibodies and vaccines (Hoffmann et al., 2021).

Up to 3 February 2022, the WHO reported > 11 million confirmed COVID-19 cases and > 147,000 deaths due to this disease in Italy (World Health Organization [WHO], 2022b). Regional reference laboratories (ISS, 2021a), such as the one of Palermo that is centrally involved in this study have played in Italy a paramount role in the detection of infected people. The Palermo institute surveys the Italian island of Sicily, which hosts a population of about 5 million inhabitants. By the end of sample gathering for this study (mid-December 2020), the number of COVID-19 cases diagnosed in Sicily was 84,835. However, for the period studied here only in seven cases the viral genome was sequenced and deposited in the GISAID public databank (EPI_ISL_2308744, EPI_ISL_2308745, EPI_ISL_2308746, EPI_ISL_2308747, EPI_ISL_2308749, EPI_ISL_3274295, and EPI_ISL_910332) (ISS, 2021b). Therefore, knowledge was scant regarding the nature of the viral lineages circulating in Sicily during the second wave of the disease.

This pilot study contributes to remediating this lack of knowledge by characterizing the variants circulating in Sicily at the end of the prevaccinal period. For achieving this goal, we have used 54 SARS-CoV-2-positive samples collected by our institute of Palermo from inhabitants of Sicily, largely in September and early October, before the beginning and in the growth phase of the second pandemic wave, and in early December 2020, when the second wave started to decline (Table 1). We searched for variants *via* sequencing of selected viral genomic regions encompassing the defining mutation sites of the B.1.177 lineage, thus being able to assess the relative prevalence of this variant. In the samples found not to belong to the B.1.177 lineage, we also analyzed a partial sequence of the *S* gene that encodes a part of the receptor-binding domain (RBD) of the S protein. This highly variable sequenced region hosts a number of key sequence changes found in SARS-CoV-2 variants, including B.1.160 and the variants of concern B.1.1.7, B.1.351, and P.1 (Gómez et al., 2021), making it appropriate for pilot searching of these variants, which in other parts of Europe began to increase their prevalence in December 2020^1^. Because of its variability, this region also appears favorable, in principle, for the detection of the novel variants.

**TABLE 1:** Sample information.

ID	Date	Ct for the targeted regions			Variant identified	Additional mutations
		*ORF1ab*	*S*	*N*		
PA57583	01/09/2020	13.72	14.91	15,42	B.1.177	
PA57891	02/09/2020	10,94	10.94	12.78	B.1.177	
PA58234	03/09/2020	11.02	11.42	13.43	B.1.177	
PA58236	03/09/2020	15,39	14.62	16,26	B.1.177	
PA58243	03/09/2020	17.15	17.34	17.96	B.1.177	
PA58968	04/09/2020	12.57	11.89	13.4	B.1.177	
PA58981	04/09/2020	13.94	13,51	15,8	B.1.177	
PA5991	04/09/2020	11,17	10.97	11.68	B.1.177	
PA59042	04/09/2020	11.95	12.02	13,33	B.1.177	
PA59067	04/09/2020	13.97	11.88	14,7	B.1.177	
PA59059	04/09/2020	22.62	22.73	25.41	Pre-existing Wuhan	
PA62148	14/09/2020	21.15	17.97	22.36	B.1.177	
PA62720	15/09/2020	11.59	11.7	12.69	B.1.177	
PA62743	15/09/2020	13.39	13.32	13.64	B.1.177	
PA65252	17/09/2020	16.66	16.57	19.02	B.1.177	
PA65276	17/09/2020	16.27	15.35	18.97	B.1.177	
PA65285	17/09/2020	13.67	13.51	15.34	B.1.177	
PA67704	23/09/2020	19.72	15.6	19.89	B.1.177	
PA67793	23/09/2020	22.97	19.39	23.46	Pre-existing Wuhan	
PA77591	15/10/2020	11.94	11.44	13.97	B.1.177	
PA80503	21/10/2020	11.67	11.81	14.93	B.1.177	
PA117525	04/12/2020	10.2	10.25	12.04	B.1.177	
PA117545	04/12/2020	11.31	11.8	13.99	B.1.177	
PA117667	04/12/2020	10.76	11.13	12.78	B.1.177	
PA117741	04/12/2020	16.8	14.71	16.79	Undetermined	G28875T (N_S201I)
PA117895	04/12/2020	13.46	11.29	15.63	B.1.177	
PA117912	04/12/2020	11.77	11.94	12.73	B.1.177	
PA118201	04/12/2020	12.21	12.38	15.79	Undetermined	G28875T (N_S201I)
PA118273	04/12/2020	10.87	11.56	12.49	B.1.177	
PA118338	04/12/2020	16.64	16.34	16.95	Undetermined	G28903T (N_M210I) C28905T (N_A211V)
PA118376	04/12/2020	11.67	11.31	12.36	Undetermined	G28875T (N_S201I)
PA118507	04/12/2020	16.02	15.09	17.31	B.1.177	
PA118573	04/12/2020	11.59	12.13	13.59	B.1.160	
PA118732	04/12/2020	11.69	11.23	11.96	B.1.177	
PA118586	04/12/2020	11.51	11.55	14.08	B.1.160	
PA118615	04/12/2020	12.07	12.54	15.29	B.1.177	
PA118625	04/12/2020	13.96	12.89	14.46	B.1.160	
PA118642	04/12/2020	12.64	12.06	14.65	B.1.177	
PA118659	04/12/2020	14.46	14.68	14.42	B.1.160	
PA118723	04/12/2020	12.22	12.22	12.57	Pre-existing Wuhan	
PA117797	04/12/2020	10.52	10.25	11.86	B.1.177	
PA118148	04/12/2020	11.58	12.47	13.14	B.1.177	
PA120227	09/12/2020	11.93	12.47	12.79	B.1.160	
PA120229	09/12/2020	11.42	11.35	11.69	B.1.160	
PA120230	09/12/2020	11.73	12.89	13.98	B.1.177	T29685C (*ORF10*)
PA120241	09/12/2020	12,34	12,11	13,89	B.1.177	T29685C (*ORF10*)
PA1120370	09/12/2020	10,74	10,83	11,48	Pre-existing Wuhan	
PA120623	09/12/2020	11,64	11,7	13,2	B.1.160	
PA120628	09/12/2020	8,25	8,86	9,76	B.1.177	
PA120636	09/12/2020	13.35	12.98	14.32	Pre-existing Wuhan	
PA120695	09/12/2020	10.74	9.54	12.84	B.1.177	
PA120704	09/12/2020	12.06	11.29	12.51	B.1.177	
PA120758	09/12/2020	11.15	12.69	14.57	B.1.177	
PA120812	09/12/2020	11.92	11,82	13,6	B.1.177	

*The columns show sample number, collection date, Ct from ORF1ab, S, and N RNA regions, variant identified, and presence of additional mutations not found in the B.1.177, B.1.160 or pre-existing Wuhan variants. Pre-existing Wuhan indicates that in all the sequenced regions (including the S region amplified in the 4th PCR reaction of Table 2) the sample conformed to the reference sequence. In line with current trends, the observation of the synonymous change in ORF10 (labeled ORF10 in the Additional mutations column) did not prevent our consideration of these samples as belonging to the B.1.177 variant revealed by the sequenced regions. In contrast, the non-synonymous mutation in the N gene (labeled in the last column as N_ preceding the amino acid change between parentheses) led us to consider the sample as having an undetermined variant.*

## Materials and Methods

### Procurement of Samples

**FIGURE 1:**
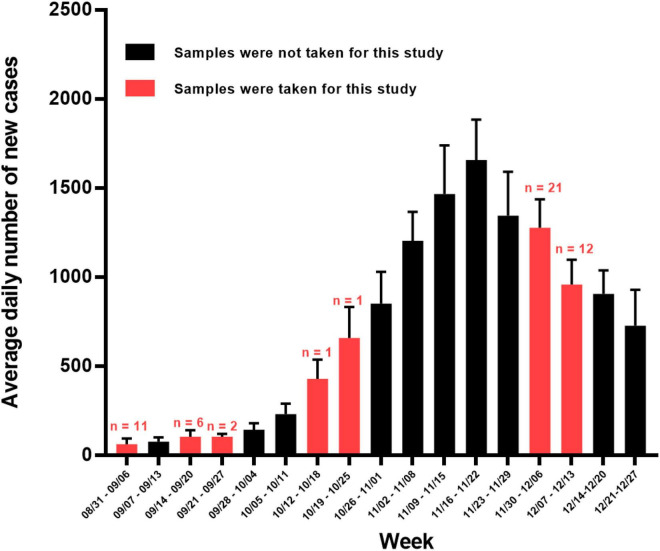
Average daily number of new SARS-CoV-2 positive cases per week in Sicily between 31 August 2020 and 27 December 2021. Red bars denote weeks in which samples were taken for this study (number of samples taken shown above red bars) whereas non-sampled weeks are colored black. Error lines show SDs. Data for Sicily were downloaded from the GitHub repository (https://github.com/pcm-dpc/COVID-19/tree/master/dati-regioni).

Between September and December 2020, a total of 20,258 nasopharyngeal swabs from individuals suspected of having COVID-19 were brought to the Virology Department at Istituto Zooprofilattico Sperimentale della Sicilia (Sicily, Italy) and were analyzed by RT-PCR for SARS-CoV-2. Positive SARS-CoV-2 amplification was obtained in 7,206 (35.57%) samples, while 11,933 (58.90%) tested negative and 1,119 (5.52%) were SARS-CoV-2 inconclusive because only one target gene for SARS-CoV-2 was positive or the Ct value for one or more targets were ≥ 37 (Ct cutoff positive value for assay targets). Some of the swab samples belonged to the migrants from many African countries and were the subject of a previous investigation (Tramuto et al., 2021). For this study, we analyzed SARS-CoV-2 positive samples collected from the Sicilian residents between September and December 2020. Table 1 summarizes the samples used, while Figure 1 shows the time of sample collection throughout the period of the second wave of COVID-19 in Sicily. For practical reasons, we were only able to examine about 50 samples (final, 54 samples). The initial part of the Results and Discussion describes the criteria used for sample selection.

### Ethical Approval

This study was conducted with the approval of the ethics committee of Cardenal Herrera CEU University, Valencia, Spain (n. CEI20/083 released on 10/09/2020), and it is in agreement with the Helsinki Declaration.

### Molecular SARS-CoV-2 Detection

This step was performed in the Virology Department at Istituto Zooprofilattico Sperimentale della Sicilia “A.Mirri” (Palermo, Italy). First, the total RNA was extracted by MagMAX™ Viral/Pathogen Nucleic Acid Isolation Kit (Applied Biosystems, Thermo Fisher Scientific, Waltham, MA, United States) employing the KingFisher Flex 96 automatic nucleic acid extractor (Thermo Fisher Scientific, Waltham, MA, United States) of QIAamp Viral RNA Mini Kit (QIAGEN, Hilden, Germany) following the instructions of the manufacturer.

Second, SARS-CoV-2 RNA detection was preliminarily performed by real-time reverse transcription polymerase chain reaction (rRT-PCR), according to the protocol of TaqPath™ COVID-19 CE-IVD RT-PCR Kit (Applied Biosystems, Thermo Fisher Scientific, Waltham, MA, United States). This multiplex assay contains three primer/probe sets specific to different SARS-CoV-2 genomic regions: *ORF1ab*, *S*, and *N* genes (TaqPath COVID-19 CE-IVD RT-PCR Kit, Applied Biosystems, Thermo Fisher Scientific, Waltham, MA, United States). The amplification was carried out by using a QuantStudio 6 Flex Real-Time PCR Systems (Applied Biosystems, Thermo Fisher Scientific, Waltham, MA, United States). Finally, once SARS-CoV-2 positivity was confirmed, RNA extracted previously from 54 positive samples was sent to the Health Sciences Faculty of UCH-CEU University (Valencia, Spain) preserved in dry ice to prevent RNA degradation.

### Molecular Variants Characterization

RNA was thawed immediately before the reverse transcription protocol. cDNA was generated by NZY First-Strand cDNA Synthesis Kit (NZYTech, Portugal) and was stored at -20^°^C. Briefly, 8 μl of each thawed RNA sample was reverse transcribed in a 20 μl reaction mixture containing 10 μl of NZYRT 2× Master Mix and 2 μl of NZYRT Enzyme Mix. The reaction mixture was incubated at 25^°^C for 10 min, at 50^°^C for 30 min followed by heat inactivation for 5 min at 85^°^C. Finally, 1 μl of NZY RNase H from *Escherichia coli* was added to remove RNA bound to cDNA and the final reaction mixture was incubated at 37^°^C for 20 min. The reverse transcribed products were stored at -20^°^C.

To detect the three specific mutations characterizing the B.1.177 variant (see section “Introduction,” and Table 2, reactions 1–3), three primer pairs were designed to amplify the targeted genomic regions by qPCR. The samples identified as “non-B.1.177 variant” (because they did not carry the full set of three specific mutations of the B.1.177 variant, see section “Introduction”) were subjected to an additional qPCR protocol (Table 2, reaction 4) for amplification, within the *S* gene, of a highly variable region which encodes a part of the RBD (Gómez et al., 2021).

**TABLE 2:** Primer pairs used in PCR amplifications to detect SARS-CoV-2 variants after partial sequencing, as detailed in the corresponding columns.

PCR reaction	Gene	Sequences of primers	Amplified region			Targeted genetic mutation	Possible SARS-CoV-2 tested variant
			Start position	End position	Size (bp)		
1	*S*	F: 5′-GGACCTTGAAGGAAAACAGG-3′ R: 5′-GAACCATTGGTAGATTTGCCA-3′	22,160	22,239	80	C22227T	B.1.177
2	*N*	F: 5′-GCAGTCAAGCCTCTTCTCGT-3′ R: 5′-TTGAACCAGCTTGAGAGCAA-3′	28,871	28,964	94	C28932T	
3	*ORF10*	F: 5′-ATTGCAACAATCCATGAGCA-3′ R: 5′-TAGGGAGGACTTGAAAGAGCC-3′	29,556	29,704	149	G29645T	
4	*S* (*RBD*)	F: 5′-CCGCATCATTTTCCACTTTT-3 R: 5′-AAACAGTTGCTGGTGCATGT-3′	22,728	23,124	397	A23063T	B.1.1.7
						A23063T G23012A G22813T	B.1.351
						A23063T G23012A A22812G	P.1
						G22992A	B.1.160
						C22879A	Cluster S_N439K

Primer pairs were designed using Primer 3^2^ to obtain a melting temperature around 60^°^C and a GC content of 40–60%, and to avoid dimerization, hybridization to unwanted sites, and the presence of secondary structures that could interfere with the amplification process. qPCR reactions were carried out by following the protocol given in the NZYSpeedy qPCR Green Master Mix (2×) (NZYTech, Portugal), which relies on SYBR green intercalation to generate the fluorescent signal. We used 2 μl of cDNA as the template in a 20-μl reaction mixture containing 10 μl of NZYSpeedy qPCR Green Master Mix (2x), 0.4 μl of 20 μM each primer, and 7.2 μl of nuclease-free water. The temperature program for all qPCR reactions was the same: (i) hot start: 2 min at 95^°^C; (ii) amplification: 40 cycles, with one cycle consisting of 5 s at 95^°^C and 30 s at 60^°^C; and (iii) melting: 30 s at 95^°^C, 30 s at 65^°^C, and 30 s at 95^°^C. Amplified DNA products were visually confirmed by agarose gel electrophoresis with fluorescent identification of bands of the expected size in the gel (Table 2). During each qPCR run, negative control using water as a template, and positive control (human sample) belonging to the B.1.177 variant were included.

### Sequencing

After qPCR amplification, the desired amplified fragments were Sanger sequenced using as sequencing primer the forward primer utilized in the amplification. Given the small size of the amplicons (Table 2), only the forward DNA sequence was determined for each sequencing reaction. DNA purification and sequencing were carried out by a core sequencing service (Genomic Department, Principe Felipe Research Centre, Valencia, Spain) in an ABI Prism 3730 automated sequencer (Applied Biosystems, Foster City, CA, United States). All sequences were subjected to BLASTN^3^ to identify related SARS-CoV-2 sequences deposited in the GenBank database. BioEdit ver. 7.2.5 software (Hall, 1999) was used for nucleotide and corresponding amino acid sequences alignment, and for analysis and calculation of the degree of identity of the retrieved sequences.

### Statistical Analysis

For the statistical analysis, R software was used (R Core Team, 2021). We divided the samples into two groups: those collected pre-December 2020 (September and October 2020) and those sampled during December 2020. A non-parametric Fisher’s exact test was used to compare the proportion of samples which belonged to the different SARS-CoV-2 variants analyzed in this study. *P*-value was calculated from 2-sided test using 0.05 as the significance level.

## Results and Discussion

The characteristics of the 54 samples analyzed in this study, including the dates of collection and molecular information derived from the present studies, are summarized in Table 1. We focused on the early part of the downslope of Sicily’s second wave (Figure 1) when the B.1.160 viral lineage was spreading through continental Europe (Hodcroft et al., 2021). For this period, we randomly chose 2 days (4 and 9 of December). From the samples collected on these 2 days, we randomly selected 33 of them obtained from unrelated individuals among those samples that had high viral loads, reflected in Ct values < 18 for the three genes examined in the diagnostic qRT-PCR (*ORF1ab*, *S*, and *N* genes, see section “Materials and Methods”). This last criterion sought to maximize success in molecular studies. To obtain insight into the variants circulating before the second wave and in the early stages of it, we included 21 additional samples in the study. Eleven of these samples were obtained from 1 to 4 September 2020, in advance of the wave; a further 8 samples were collected on 12–23 September 2020, closer to the beginning of the wave; and single samples, each from 15 and 21 October 2020, were from the early phase of the wave. Due to the paucity of cases and samples during the month of September, four samples for this month (one taken 4 September and the other three in the second half of the month) had at least one Ct value > 18 (although all Ct values were < 26) (Table 1).

**FIGURE 2:**
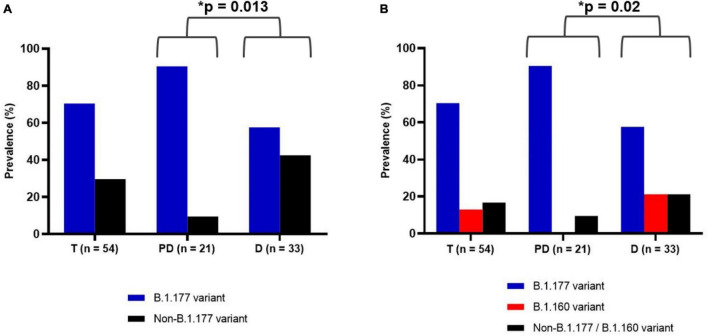
Prevalence of SARS-CoV-2 variants among the 54 samples during the whole assayed period of this study (T), before December 2020 (pre-December group, PD) and in December 2020 (December group, D). The number of samples is given in the abscissa. **(A)** Prevalence of B.1.177 variant (blue) and non-B.1.177 variant (black). **(B)** Prevalence of B.1.177 (blue), B.1.160 (red), and non-B.1.177/B.1.160 variant (black). The asterisk indicates a statistically significant difference (*p*-value given by the Fisher’s exact test, see section “Materials and Methods”).

For each sample, we amplified and sequenced three SARS-CoV-2 genomic regions (Table 2, PCR reactions 1–3) encompassing the three specific nucleotide mutations of the B.1.177 lineage, C22227T, C28932T, and G29645T, mapping to the *S*, *N*, and *ORF10* genes, respectively. Of the 54 samples, 38 (70.4%) corresponded to the B.1.177 variant (Figure 2A), two of which hosted a synonymous nucleotide change in the sequenced region of the *ORF10* gene (Table 1). We compared the relative prevalence of this variant before and at the start of the second wave with that during the downslope of the wave (Figure 1). With this goal in mind, we distributed the samples into two groups, the “pre-December 2020” group, which included the 21 samples collected in September and October of 2020; and the “December 2020” group, which included the 33 samples gathered in December 2020 (Figure 2). While 90.47% (19/21) of the samples in the pre-December 2020 group corresponded to the B.1.177 variant, this variant was only found in 57.6% (19/33) of the samples in the December 20 group (Figure 2A). A Fisher’s exact test was carried out to statistically compare the relative prevalence of the B.1.177 variant among the samples in these two groups. A *p* = 0.013 confirmed that the difference in the relative prevalence of this variant before December 2020 and in December 2020 was statistically significant.

To identify which SARS-CoV-2 variants appeared alongside the lowering in the relative prevalence of B.1.177 in the downslope of the second wave, we amplified and sequenced the central part of the genomic region that encoded the RBD part of the S protein (see section “Materials and Methods” and Table 2) in the 16 non-B1.177 viral samples. The amplified region was chosen because it is highly variable and its sequence would allow the identification of the B.1.1.7, B.1.351, and P.1 lineages (Bayarri-Olmos et al., 2021) and the B.1.160 lineage and a smaller cluster defined by the N439K S protein mutation (Hodcroft et al., 2021). Most of these variants considerably increased in prevalence in Europe around December 2020 (see text footnote 1). Through alignment with the SARS-CoV-2 reference genome (GenBank Accession Number: NC_045512.2), we detected that the only two non-B.1.177 sequences in the pre-December 2020 group (9.52%, 2/21) were identical in this region to the reference genome. On the other hand, 7 of the 14 non-B.1.177 samples gathered in the December 2020 group (21.2%, 7/33), contained the G22992A non-synonymous mutation. This mutation corresponds to the specific S477N substitution in the S protein of the B.1.160 variant (Figure 2B). Therefore, we were able to distribute the 54 samples in three sets: set (1) B.1.177 variant (*n* = 38), set (2) B.1.160 variant (*n* = 7), and set (3) non-B.1.177/B.1.160 variants (*n* = 9), of which 5 and 4 represented, respectively, the pre-existing Wuhan sequence and undetermined variants. The differences in the proportions of samples in these three sets in the pre-December 2020 and December 2020 groups were statistically significant (*p* = 0.020) (Figure 2B).

Interestingly, six samples presented additional mutations in the *ORF10* or *N* gene (Table 1). We already mentioned two of these samples (PA120230 and PA120241, Table 1), which contained a synonymous mutation in *ORF10* in addition to the three specific mutations that characterize the B.1.177 variant. The other four samples belonged to set (3), non-B.1.177/B.1.160, and represented previously undetermined variants. The RBD mutations found in these samples were proven by BLASTN analysis (see text footnote 3) to have been previously detected multiple times, although they had not been used to define or to participate in defining any lineage.

Our finding of the predominance of the B.1.177 variant and the timing of the appearance of the B.1.160 variant and of undetermined variants replicates the findings in other parts of Europe in approximately the same period (Hodcroft et al., 2021; see text footnote 1). This indicates that the insular character does not result in a particular pattern of variants reflecting isolation. Yet, further exploration of the undetermined variants should be implemented through a whole-genome sequencing approach to test the possibility that these variants could be “private” to Sicily and novel, arising locally or *via* immigration from other continents (largely Africa). The discussion on whether the slightly increased affinity for the ACE2 receptor of the S protein found in the B.1.160 viral variant (Chen et al., 2020), or the relaxation of restrictions in mobility throughout Europe caused the increase in the B.1.160 viral variant remains unanswered for Europe, and for Sicily. On this island, heavy business and touristic travel in and out of it may be a reason for the similarity of Sicily to the remainder of the continent in terms of variants prevalence. However, our failure to identify in any sample the B.1.1.7, B.1.351, or P.1 variant suggests some delay in the colonization of Sicily by these lineages, which present clear differential traits concerning viral biology and ability to spread across the population, as best exemplified in the increased transmissibility of B.1.1.7 (Davies et al., 2021). Further studies focusing on later periods in the pandemic are needed to analyze the spread of these novel variants in Sicily. These studies would clarify if, as in many other regions of Europe (Funk et al., 2021), these variants became predominant in late December 2020 and January 2021, a period of resurgence of the number of cases that could be equated to a second wave within the second wave. In any case, our pilot study suggests that in the period studied no SARS-CoV-2 variant of significantly higher transmissible potential than the consensus one emerged in Sicily.

## Data Availability Statement

The datasets presented in this study can be found in online repositories. The names of the repository/repositories and accession number(s) can be found below: https://www.ncbi.nlm.nih.gov/genbank/, OM510944; https://www.ncbi.nlm.nih.gov/genbank/, OM510945; https://www.ncbi. nlm.nih.gov/genbank/, OM510946; https://www.ncbi.nlm.nih. gov/genbank/, OM510947; https://www.ncbi.nlm.nih.gov/genbank/, OM510948; https://www.ncbi.nlm.nih.gov/genbank/, OM510949; https://www.ncbi.nlm.nih.gov/genbank/, OM51 0950; https://www.ncbi.nlm.nih.gov/genbank/, OM510951; and https://www.ncbi.nlm.nih.gov/genbank/, OM510952.

## Ethics Statement

The studies involving human participants were reviewed and approved by the Bioethical Committee of Universidad Cardenal Herrera CEU. Written informed consent for participation was not required for this study in accordance with the national legislation and the institutional requirements.

## Author Contributions

AG, CR-G, VR, GP, and EM conceived the study. FG, GP, AP, and MT performed molecular SARS-CoV-2 detection in all the samples. MP-B did the molecular variants characterization. CR-G, EM, and MP-B analyzed the results. CS, VV, MP-O, and MP-B did the epidemiological analysis. CS, MP-B, EM, GP, FG, AG, VR, and CR-G analyzed the data and were responsible for writing the manuscript. All authors contributed to this last task, making substantial intellectual contributions, and having read, corrected, and approved the manuscript.

## Conflict of Interest

The authors declare that the research was conducted in the absence of any commercial or financial relationships that could be construed as a potential conflict of interest.

## Publisher’s Note

All claims expressed in this article are solely those of the authors and do not necessarily represent those of their affiliated organizations, or those of the publisher, the editors and the reviewers. Any product that may be evaluated in this article, or claim that may be made by its manufacturer, is not guaranteed or endorsed by the publisher.

## Footnotes

1
https://covariants.org

2
https://primer3.ut.ee/

3
http://blast.ncbi.nlm.nih.gov
